# Homogeneous continuous flow nitration of *O*-methylisouronium sulfate and its optimization by kinetic modeling

**DOI:** 10.3762/bjoc.20.205

**Published:** 2024-09-24

**Authors:** Jiapeng Guo, Weike Su, An Su

**Affiliations:** 1 Key Laboratory of Pharmaceutical Engineering of Zhejiang Province, Key Laboratory for Green Pharmaceutical Technologies and Related Equipment of Ministry of Education, Collaborative Innovation Center of Yangtze River Delta Region Green Pharmaceuticals, Zhejiang University of Technology, Hangzhou, 310014, P. R. China; 2 State Key Laboratory Breeding Base of Green Chemistry-Synthesis Technology, Key Laboratory of Green Chemistry-Synthesis Technology of Zhejiang Province, College of Chemical Engineering, Zhejiang University of Technology, Hangzhou, Zhejiang 310014, China

**Keywords:** continuous flow, kinetic modeling, nitration, reaction optimization, static mixer

## Abstract

Nitration of *O*-methylisouronium sulfate under mixed acid conditions gives *O*-methyl-*N*-nitroisourea, a key intermediate of neonicotinoid insecticides with high application value. The reaction is a fast and highly exothermic process with a high mass transfer resistance, making its control difficult and risky. In this paper, a homogeneous continuous flow microreactor system was developed for the nitration of *O*-methylisouronium sulfate under high concentrations of mixed acids, with a homemade static mixer eliminating the mass transfer resistance. In addition, the kinetic modeling of this reaction was performed based on the theory of NO_2_^+^ attack, with the activation energy and pre-exponential factor determined. Finally, based on the response surface generated by the kinetic model, the reaction was optimized with a conversion of 87.4% under a sulfuric acid mass fraction of 94%, initial reactant concentration of 0.5 mol/L, reaction temperature of 40 °C, molar ratio of reactants at 4.4:1, and a residence time of 12.36 minutes.

## Introduction

The demand for high-quality insecticides is increasing as the world’s food crisis intensifies due to the changes in the natural environment and ongoing geopolitical crises [1]. *O*-Methyl-*N*-nitroisourea (NIO) is a pivotal pesticide intermediate in the preparation of nitroguanidine derivatives, which are the raw material for highly effective and non-toxic neonicotinoid insecticides, such as dinotefuran and clothianidin [2–4]. Currently, the industrial production of *O*-methyl-*N*-nitroisourea usually involves the nitration of *O*-methylisouronium sulfate (IO) with a mixture of sulfuric acid (H_2_SO_4_) and nitric acid (HNO_3_) in a batch reactor [3]. The reaction is a typical aliphatic nitration, which is fast and highly exothermic, requiring low reaction temperatures. In addition, the safety hazard of this reaction is increased by using concentrated nitric and sulfuric acids. Therefore, it is necessary to modify the nitrification reaction process of *O*-methylisouronium sulfate to improve the reaction efficiency and intrinsic safety.

In recent years, continuous flow microreactors have been recognized due to their excellent mass and heat transfer performance, precise control over reaction parameters, and intrinsic safety [5–8]. Guo et al. constructed a continuous flow microsystem for *o*-xylene nitrification and proved the process safety of by the adiabatic temperature rise of the nitrification reaction and the characteristic heat transfer time of the microreactor [9]. The residence time of the microreactor was reduced by an order of magnitude and the volumetric mass transfer coefficient was increased by several orders of magnitude compared with that of a conventional stirred-tank reactor. Jin et al. developed a continuous flow microreactor system for the non-homogeneous nitrification of nitrobenzene using mixed acids [10]. The reaction time and temperature were reduced from >2 h and 80 °C in industrial operation to 10 min and 65 °C in the microreactor with high conversion and selectivity. Since *O*-methylisouronium sulfate can be dissolved in high concentrations of sulfuric acid, it is expected to construct a homogeneous continuous flow nitrification system, leading to better elimination of the effects of mass and heat transfer [11].

Kinetic modeling is a classical approach to chemical reaction optimization, where the effects of various reaction parameters on the results are effectively quantified by mathematical formulas, thus providing an efficient guide to optimize reaction conditions [12]. Taylor et al. [13] and Bures et al. [14] have performed kinetic modeling with data collected from continuous flow systems with automated platforms. Yao et al. constructed a kinetic model on thermal dissociation and oligomerization of dicyclopentadiene (DCPD) in a continuous flow microreactor [15]. Where cyclopentadiene was the target intermediate formed by the thermal dissociation of dicyclopentadiene, cascade oligomerization was a side reaction to be avoided. Based on the deep understanding of the kinetic differences between thermal dissociation and oligomerization, the residence time and temperature were designed rationally to improve the yield of cyclopentadiene. Since NO_2_^+^ is the actual substance that plays a role in the nitrification process [16], kinetic modeling based on the concentration of NO_2_^+^ is essential for the understanding of the nitrification mechanism and optimization of the reaction. Luo et al. have carried out extensive research on this topic and obtained accurate kinetic data for the nitration of chlorobenzene [17], *o*-nitrotoluene [18], and *p*-nitrotoluene [19] by constructing a homogeneous continuous flow reaction system. Therefore, it is feasible to model homogeneous nitrification and optimize the reaction in a continuous flow system based on NO_2_^+^.

An important prerequisite for kinetic modeling is the elimination of issues related to mass and heat transfer. The effect of mass transfer resistance is greater for highly viscous reaction systems, especially at higher reactant concentrations. It is still difficult to eliminate the mass transfer effect using conventional microreactors, leading to errors in the determination of nitration kinetics. Therefore, more efficient mixers are needed to overcome the effects of mass and heat transfer. According to the mixing principle, there are active mixers and passive mixers. Passive mixers do not require overly complex equipment and external energy inputs and are extensively used in continuous flow reactions [20–21]. Passive mixers enhance the passive mixing of the liquid–liquid two-phase mass transfer process on a microscopic scale, mainly by optimizing the microchannel geometry [22], addition of in-channel obstacles, etc. [23–25]. Santana et al. designed an efficient fluid mixer "Elis" consisting of internal walls and circular obstacles. This static mixer achieves efficient mixing in a wide range of Reynolds numbers at the micro- and milliscale. However, many static mixer designs are structurally complex and require the use of 3D printing technology to aid in their manufacture, which is more expensive to use. Kilcher et al. investigated in detail the efficient mixing of organic phases (cyclopentadiene, 1,2-dichloroethane, and MeBu_3_NCl) and aqueous phases (30% NaOH) and optimized it by the use of a simple homemade “PTFE Raschig ring static mixer” (RRSM). The RRSM is simple in structure, easy to fabricate, inexpensive for many flow reaction systems, and has a promising application.

**Figure 1:**
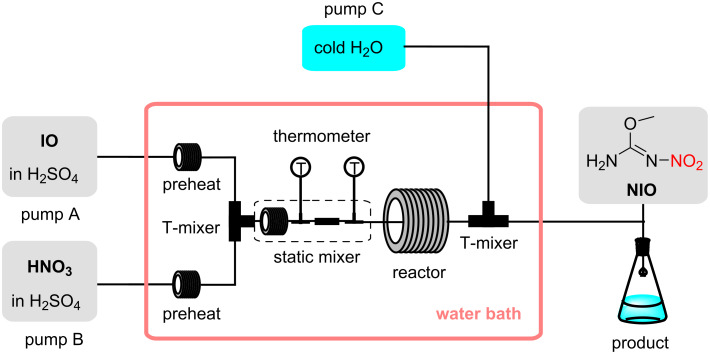
The schematic diagram of the continuous flow microreactor system.

In this work, we constructed a continuous flow microreactor system to determine the kinetic parameters of IO nitration, which allows precise control of temperature and residence time (Figure 1). Due to the high viscosity of the reaction system, a simple and practical static mixer was designed to eliminate the effect of mass transfer on the kinetic measurements and validated experimentally. We developed a kinetic model for the nitration of *O*-methylisouronium sulfate and optimized the reaction conditions for conversion rates, which is crucial for theoretical significance and practical value for process optimization.

## Results and Discussion

**Scheme 1:**
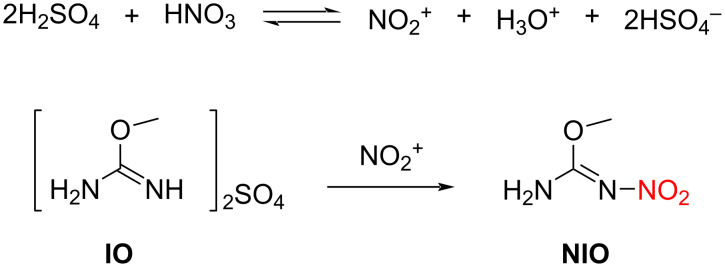
Nitration of IO with mixed acid.

In this section, we perform kinetic modeling for the continuous flow synthesis of NIO from IO and mixed acid (Scheme 1). The reaction was then optimized by kinetic modeling.

### Prescreening experiments

The solubility of IO in H_2_SO_4_ is critical in ensuring the smooth progression of the nitration reaction within a homogeneous system. Given the strongly exothermic nature of this reaction, an excessively high concentration of IO can lead to an overproduction of heat, thereby elevating the associated risks. In contrast, a concentration that is too low may fall beneath the detection threshold, compromising the reliability of the experimental data. To strike a balance, the initial concentration of IO was set to 0.5 mol/L in the reaction mixture. In addition, the effect of the molar ratio between the two reactants was examined. As shown in Figure S1 in Supporting Information File 1, the conversion of IO gradually increased as the molar ratio of HNO_3_ elevated. The molar ratio of HNO_3_ was established at 4.4 equiv, a value chosen to optimize both conversion and atom efficiency.

### Effect of two types of mixing equipment

**Figure 2:**
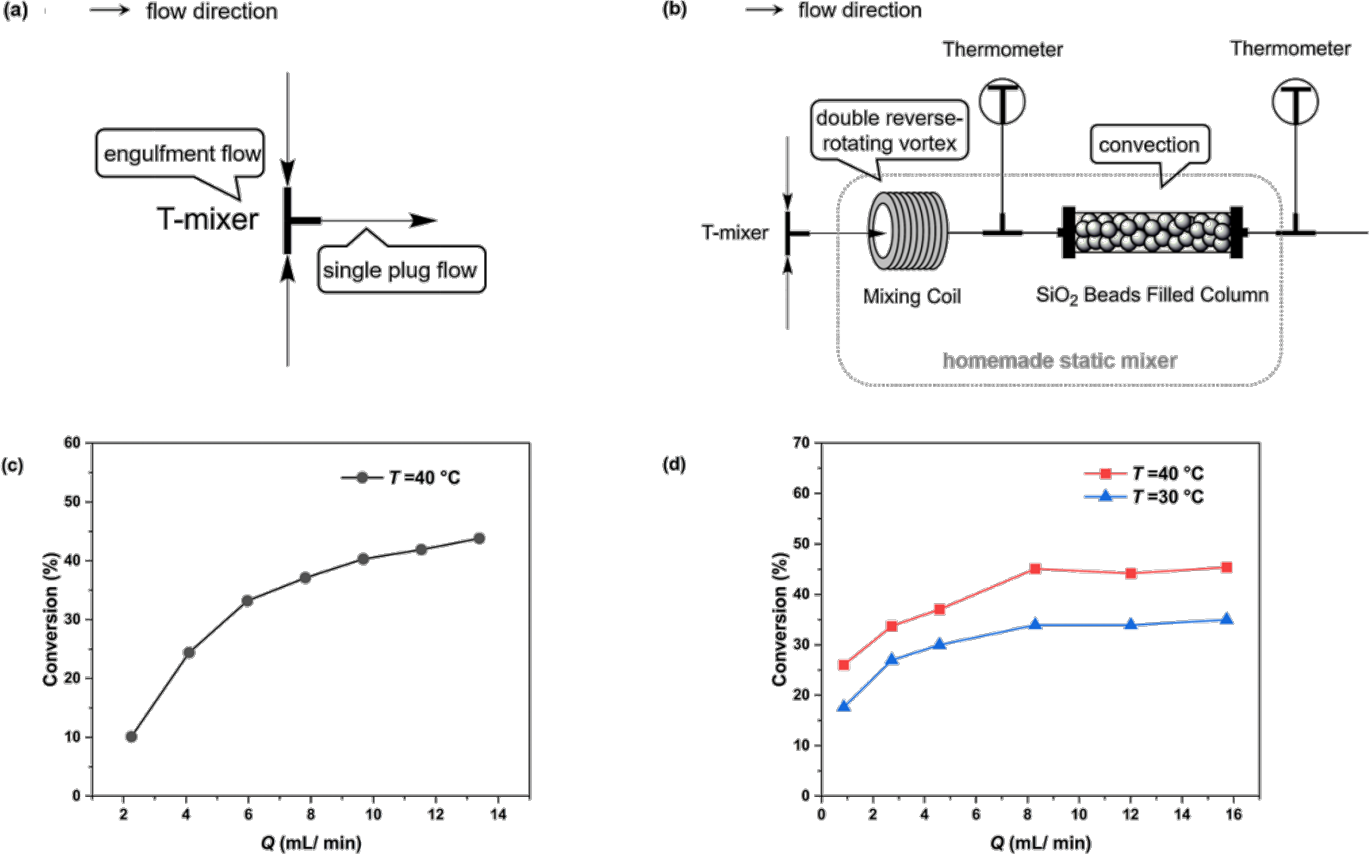
Two mixing setups: (a) a T-mixer and (b) a T-mixer combined with a homemade static mixer, and the effect of the two mixing setups on the mixing process; (c) the T-mixer and (d) the T-mixer plus the homemade static mixer effect of flow rate on conversion. Reaction conditions: H_2_SO_4_ mass fractions = 98%, reaction temperature *T* = 40 °C, residence time *t* = 2 min, initial concentration of reactants *c*_IO_ = 1 mol/L, *c*_HNO3_ = 4.4 mol/L.

Upon achieving homogeneous nitration conditions, our next objective was to eliminate the influence of mass transfer. We assessed the impact of flow rate on the reaction conversion under two distinct mixing scenarios (Figure 2a and 2c). The assessments were performed with reaction temperatures at 30–40 °C to eliminate the impact of the high viscosity of sulfuric acid [26]. Figure 2a illustrates the scenario employing solely a T-mixer and Figure 2c shows the effect of flow rate on the conversion under this setup. Even when the flow rate was escalated to 14 mL/min, the conversion failed to stabilize at a plateau, suggesting that mass transfer limitations had not been fully addressed. Conversely, with the addition of our homemade static mixer which consists of a 1/16-inch mixing coil and a SiO_2_ beads-filled column (Figure 2b), the conversion rate plateaued once the total flow rate surpassed 8 mL/min (Figure 2d), suggesting the elimination of mass transfer limitations. The improved mixing efficiency can be attributed to the mixer’s design features, such as its double reverse rotating vortex [27–28], large specific surface area [29], and the incorporation of obstacles within the flow channel [30–31].

### Determining reaction orders

**Figure 3:**
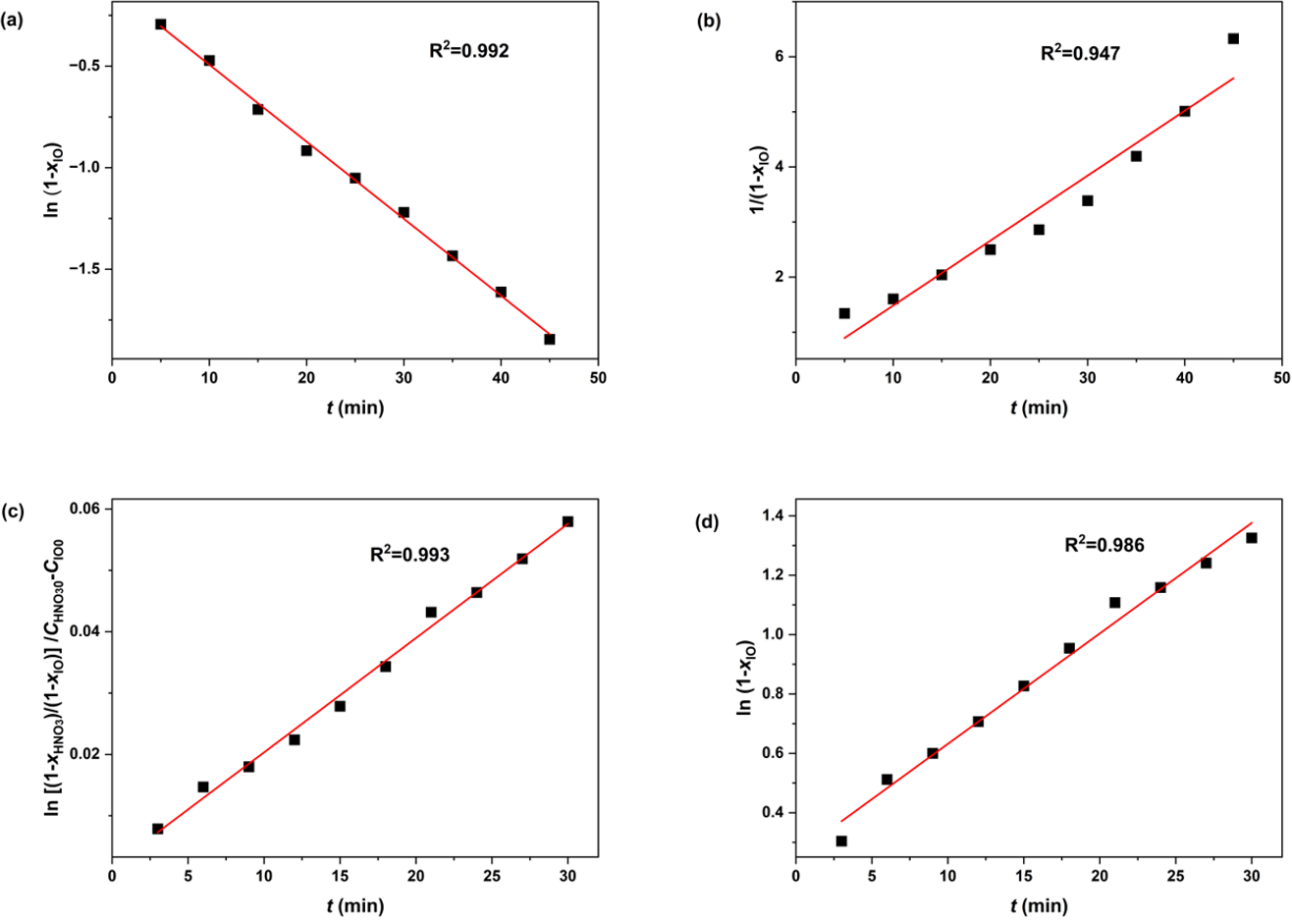
Determination of the number of reaction orders. a) ln(1−*x*_IO_) versus *t*; b) 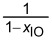 versus *t*; c) ln(1−*x*_IO_) versus *t*; d) 
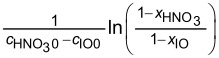
 versus *t*. Reaction conditions for determining IO’s reaction order: H_2_SO_4_ mass fractions = 98%, reaction temperature, *T* = 0 °C; initial concentration of reactants in the reaction mixture: *c*_IO0_ = 1 mol/L, *c*_HNO30_ = 15 mol/L. Reaction conditions for determining HNO_3_’s reaction order: reaction temperature, *T* = 0 °C; initial concentration of reactants in the reaction mixture: *c*_NIO0_ = 1 mol/L, *c*_HNO30_ = 4.4 mol/L.

The reaction orders for IO and HNO_3_ were determined in the continuous flow microreactor system, and the results are shown in Figure S2 of Supporting Information File 1. The initial concentration of HNO_3_ was set at a level significantly higher (14 times greater) than that of IO. This approach allowed for the assumption that the concentration of HNO_3_ remained constant throughout the reaction, enabling the conversion of the rate constant to *K*_β_ (Equation 1). The relationship between reaction time and the conversion of IO was analyzed according to the first-order (Equation 2) and second-order (Equation 3) reaction kinetics, where *x*_IO_ represented the conversion of IO and *t* denoted the reaction time. The outcome of these fittings is presented in Figure 3a for first-order and Figure 3b for second-order. Notably, the higher R^2^ observed in Figure 3a compared to Figure 3b suggests that the reaction of IO follows first-order kinetics.


[1] $$-\frac{dc_{\text{IO}}}{dt}=kc_{\text{IO}}^{\;\alpha }c_{{\text{HNO}}_{3}}^{\;\beta }\approx K_{\beta }c_{\text{IO}}^{\;\alpha }$$

[2] $$\ln(1-x_{\text{IO}})=-K_{\beta }t$$

[3] $$\frac{1}{1-x_{\text{IO}}}=1-K_{\beta }t$$

Given that the reaction order of IO was determined to be 1, Equation 1 was subsequently transformed into Equation 4. As nitration reactions are predominantly second-order, we explored the potential for the reaction order of HNO_3_ (β) to be either 0 or 1 by fitting the reaction data to Equation 5 and Equation 6, respectively.


[4] $$-\frac{dc_{\text{IO}}}{dt}=kc_{\text{IO}}c_{{\text{HNO}}_{3}}^{\;\beta }$$

[5] $$\ln(1-x_{\text{IO}})=-Kt$$

[6] $$\frac{1}{c_{{\text{HNO}}_{\text{3 0}}}-c_{\text{IO 0}}}\ln\left(\frac{1-x_{{\text{HNO}}_{3}}}{1-x_{\text{IO}}}\right)=-Kt$$

The fitting results, as depicted in Figure 3c for β = 0 and Figure 3d for β = 1, revealed that R^2^ for the latter scenario (R^2^ = 0.993) was higher than that for the former (R^2^ = 0.986). This outcome indicates that the reaction order of HNO_3_ is also 1, which transforms Equation 4 into Equation 7.


[7] $$-\frac{dc_{\text{IO}}}{dt}=kc_{\text{IO}}c_{{\text{HNO}}_{3}}$$

Also, with M=cHNO30cIO0, Equation 6 can be rewritten to Equation 8.


[8] $$\ln\left[\frac{M-x_{\text{IO}}}{M(1-x_{\text{IO}})}\right]=(M-1)c_{\text{IO}0}kt$$

After the reaction order being determined, the rest of the experiments were conducted in the continuous flow reactor and *t* in Equation 8 refers to the residence time.

### Determining the apparent reaction kinetics

**Figure 4:**
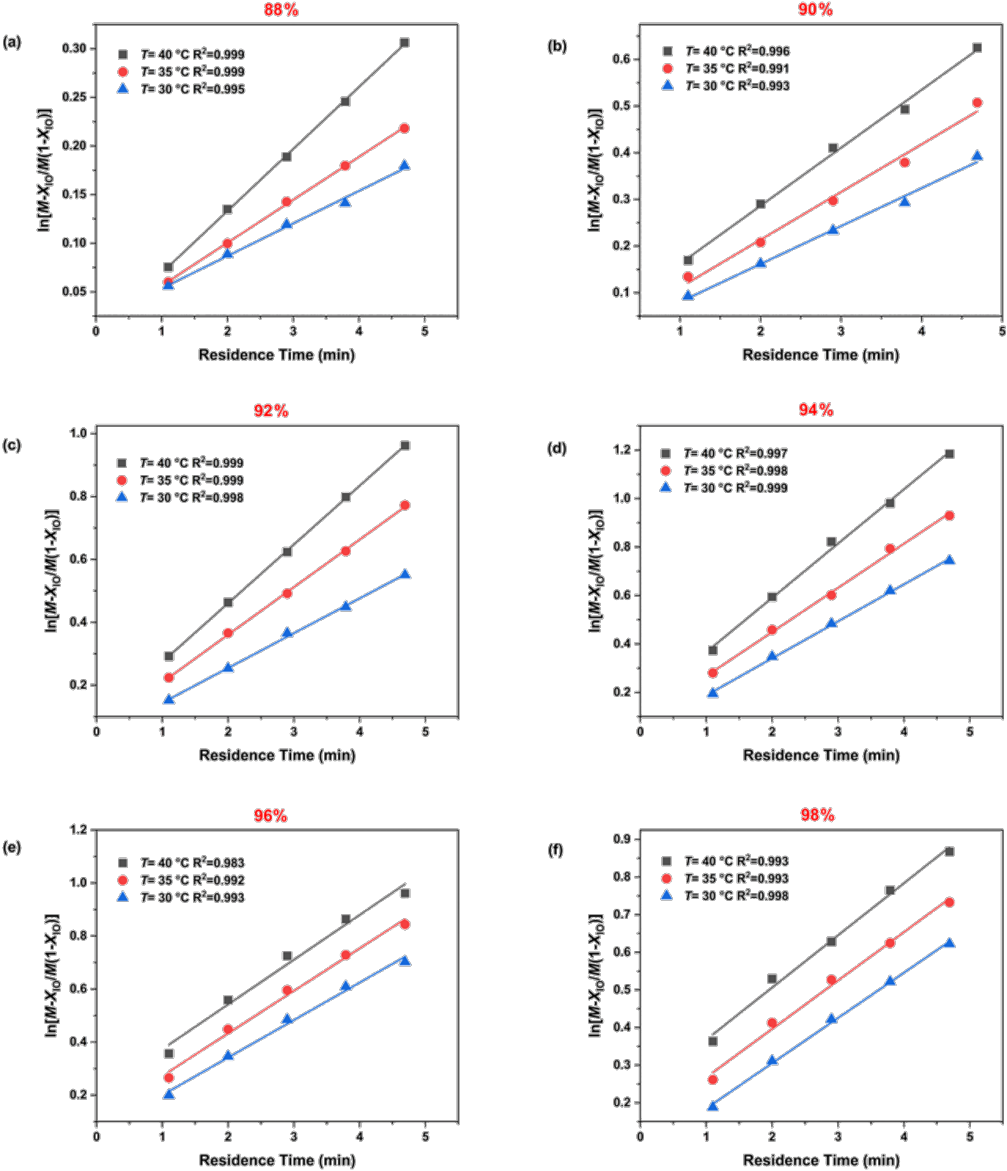
Determination of (M−1)*c*_IO0_*k* at different temperatures and H_2_SO_4_ mass fractions. (a) 88% H_2_SO_4_, (b) 90% H_2_SO_4_, (c) 92% H_2_SO_4_, (d) 94% H_2_SO_4_, (e) 96% H_2_SO_4_, and (f) 98% H_2_SO_4_.

The variation in the conversion of IO (*x*_IO_) as the function of time (*t*) at different temperatures (30 °C, 35 °C, 40 °C) and H_2_SO_4_ mass fractions (88%, 90%, 92%, 94%, 96%, and 98%) is depicted in Figure S3 in Supporting Information File 1 and subsequently modeled using Equation 8. The fitting results shown in Figure 4 exhibit robust linear correlations (R^2^ > 0.99), facilitating the calculation of rate constants based on the slopes of these lines across the varied temperatures and H_2_SO_4_ concentrations. Table 1 indicates that the reaction rate constants escalate with increasing H_2_SO_4_ mass fraction, which aligns with the findings from previous studies on mixed acid-catalyzed nitration reactions [32–33]. However, the data also reveal a decline in rate constants when the H_2_SO_4_ mass fraction exceeds 94%, suggesting a complex interaction at higher acid concentrations.

**Table 1:** Values of *k* for different H_2_SO_4_ mass fractions and at different temperatures.

Mass fraction of H_2_SO_4_ (wt %)	*k* × 10^2^ (L/mol/s)		

	30 °C	35 °C	40 °C

88	2.26	2.98	4.31
90	5.51	6.91	8.40
92	7.48	10.2	12.6
94	10.3	12.3	15.1
96	9.56	10.8	11.4
98	8.13	8.70	9.37

### Determining the intrinsic reaction kinetics

Given the strong correlation between the observed HNO_3_-based reaction rate constant and the H_2_SO_4_ mass fraction, intrinsic reaction constants independent of H_2_SO_4_ concentrations were determined to study the intrinsic kinetics of the reaction. Previous research has established that the relationship between the apparent and intrinsic kinetics of nitrification can be described by Equation 9 [17,19].


[9] $$\mathrm{lg}k=\mathrm{lg}\left(\frac{c_{{\text{NO}}_{2}^{\;+}}}{c_{{\text{HNO}}_{\text{3}}}}\right)+nM_{c}+\mathrm{lg}k_{0}$$

where *k*_0_ is the intrinsic rate constant only based on NO_2_^+^ and independent of sulfuric acid concentration [34], *n* is a thermodynamic parameter related to the type of compound, and *M*_c_ is the activity coefficient function introduced in the next section.

By shifting the terms in Equation 9, Equation 10 can be obtained as:


[10] $$\mathrm{lg}k-\mathrm{lg}\left(\frac{c_{{\text{NO}}_{2}^{\;+}}}{c_{{\text{HNO}}_{\text{3}}}}\right)=nM_{c}+\mathrm{lg}k_{0}$$

Therefore, by plotting


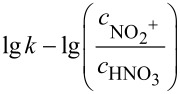


as the vertical coordinate and *M*_c_ as the horizontal coordinate, the values of *n* and *k*_0_ can be obtained from the slope and intercept of the resulting fitting line. Since the values of *M*_c_ and


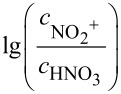


change with the change in temperature and sulfuric acid mass fraction, we determined the values of *M*_c_ and


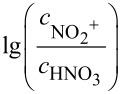


according to the method proposed by Luo et al. [17,19]. As the ranges of sulfuric acid concentrations and temperature in our study were different from Luo et al.’s study, recalculations were required to obtain the values of *M*_c_ and


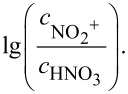


#### Determination of *M*_c_ values

The value of *M*_c_ can be calculated using Equation 11 and Equation 12. Equation 11 [35] was employed to predict *M*_c_ at various H_2_SO_4_ concentrations at 298 K, specifically when the H_2_SO_4_ concentrations were between 15.2 and 18.4 mol/L. By fitting the predicted data, *M*_c_ as a function of the H_2_SO_4_ concentration at a given temperature was determined (Figure S4 in Supporting Information File 1). In addition, the values of *M*_c_ for different sulfuric acid concentrations at a given temperature can be obtained by substituting the corresponding temperature into Equation 12, as first introduced by Marziano et al.


[11] $$\begin{array}{c} -M_{c}(\text{298 K})=2.16\times 10^{-4}c_{{\text{H}}_{2}{\text{SO}}_{4}}^{\;5}-1.27\times 10^{-2}c_{{\text{H}}_{2}{\text{SO}}_{4}}^{\;4}\\ +0.28c_{{\text{H}}_{2}{\text{SO}}_{4}}^{\;3}-2.73c_{{\text{H}}_{2}{\text{SO}}_{4}}^{\;2}+10.6c_{{\text{H}}_{2}{\text{SO}}_{4}} \end{array}$$

[12] $$M_{c}(T)=M_{c}(\text{298 K})\left[\frac{200}{T}+0.3292\right]$$

#### Determination of lg(*c*_NO2+_/*c*_HNO3_) values

Since NO_2_^+^ is the actual reactive species in the nitration reaction, an accurate estimation of its concentration is essential for the study of intrinsic kinetics. Based on the values of 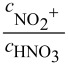, reported in previous studies for different temperatures and sulfuric acid concentrations [36–38], the mass fraction of sulfuric acid was plotted against


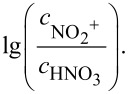


**Figure 5:**
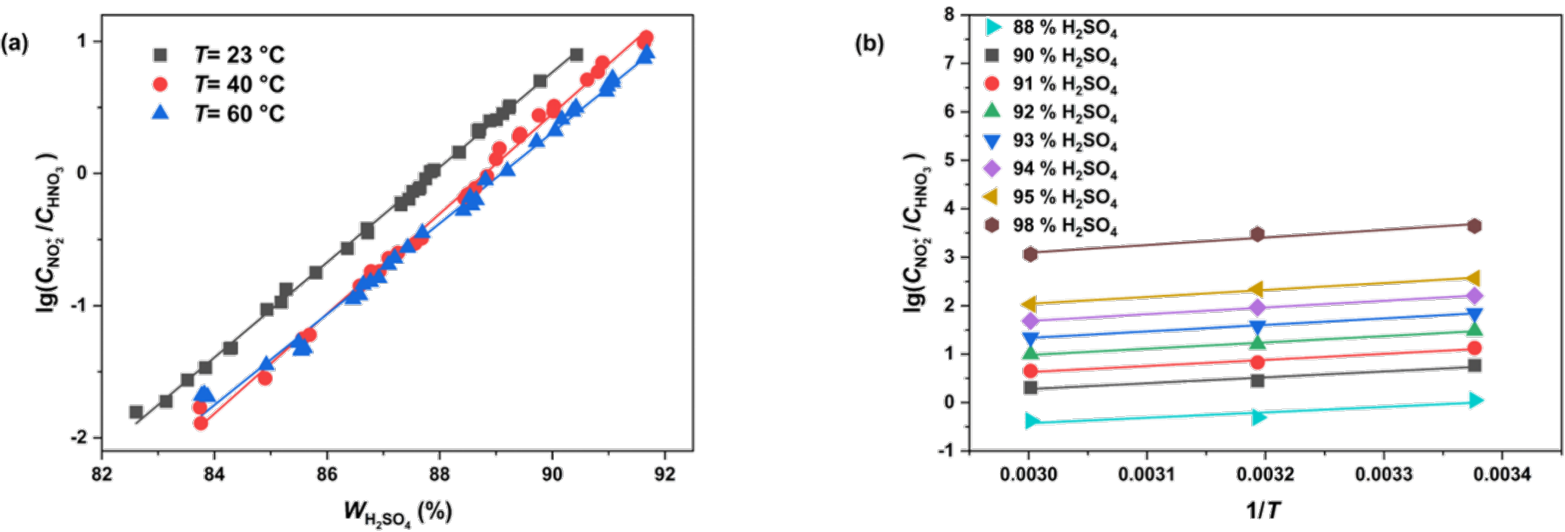
Variations and fitting of as a function of a) the mass fraction of H_2_SO_4_ at 23 °C, 40 °C, and 60 °C and b) 1/*T* at different H_2_SO_4_ concentrations 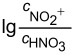.

The fitting results shown in Figure 5a exhibit robust linear correlations, enabling the calculation of


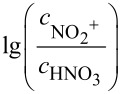


at temperatures of 23 °C, 40 °C, and 60 °C. Subsequently, by plotting


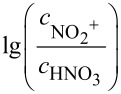


versus 1/*T*, a series of fitted curves for the studied range of sulfuric acid concentrations (88–98 wt %) can be obtained, as shown in Figure 5b. Thus, the values of


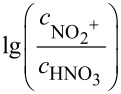


at different sulfuric acid concentrations and temperatures can be determined.

#### Determination of intrinsic kinetic parameters

**Figure 6:**
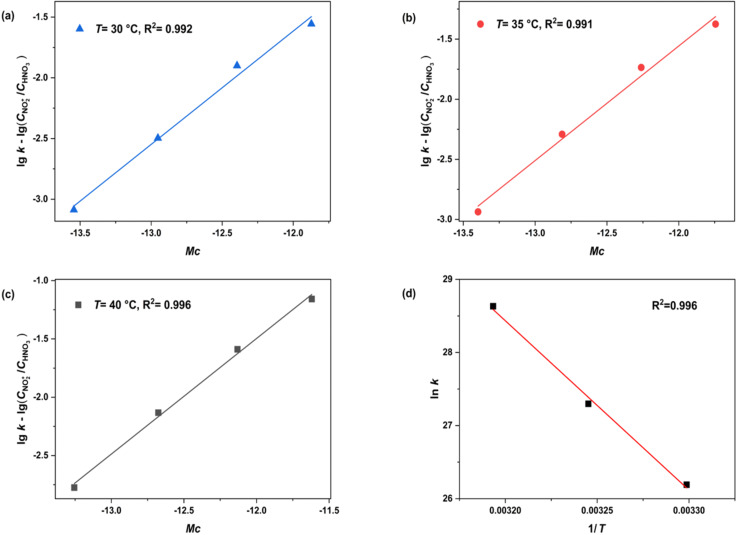
Determination of thermodynamic parameters *n* and *k*_0_ and determination of the activation energy and pre-exponential factors.

With


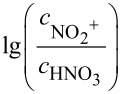


and *M*_c_ at different conditions determined in Figure 5,


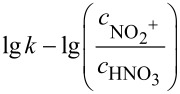


was plotted against *M*_c_ at different temperatures (Figure 6a–c), and fitting these data into Equation 10 leads to (R^2^ > 0.99). The values of *k*_0_ and *n* at different temperatures are shown in Table 2. The value of *k*_0_ increases with increasing temperature and the value of *n* remains almost constant with temperature, which is consistent with the results reported in previous studies for other mixed acid-catalyzed nitration reactions [17,39].

**Table 2:** Values of *n* and lg*k*_0_ at different temperatures.

Temperature (°C)	*n*	lg*k*_0_

30	1.0764	11.3749
35	1.1127	11.8556
40	1.1577	12.4352

According to the values of *k*_0_ at different temperatures, the activation energy for the electrophilic attack of NO_2_^+^ on the IO can be calculated by the Arrhenius equation:


[13] $$\ln k_{0}=-\frac{E_{\text{a}}}{RT}+\ln A$$

where *R* is the molar gas constant and *T* denotes the temperature in Kelvin, and *E*_a_ and *A* are the activation energy and pre-exponential factors for the IO nitration.

By fitting ln*k*_0_ versus 1/*T* into Equation 13 (Figure 6d), the values of *E*_a_ and ln*A* were determined (Table 3).

**Table 3:** Values of the pre-exponential factor and activation energy.

Factors	*E*_a_ (kJ/mol)	ln*A*

values	192.57	102.55

### The synergic effect of temperature and sulfuric acid concentration on the apparent kinetics

As discussed above, the apparent rate constant is determined by three components,


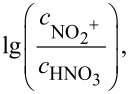


**Figure 7:**
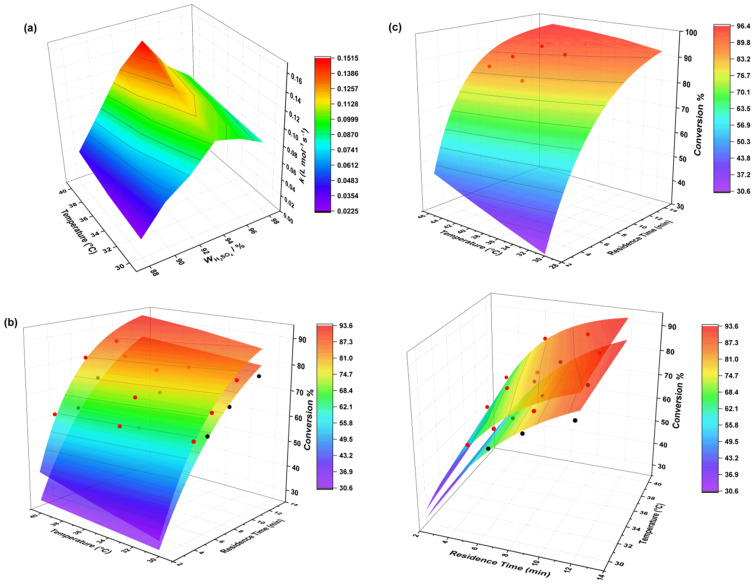
a) The value of apparent rate constant *k* at various H_2_SO_4_ mass fractions and different temperatures. b) Validation of reaction kinetic models with different mass fractions of sulfuric acid (from top to bottom: the theoretical response surfaces and experimental values are shown for sulfuric acid mass fractions of 94% and 98%, respectively). c) Response surface of the kinetic model under optimized conditions and experimental results. The red dots show the experimental values for a sulfuric acid mass fraction of 94% and the black dots show the experimental values for a sulfuric acid mass fraction of 98%.

*nM*_c_, and lg*k*_0_. First, the intrinsic rate constant *k*_0_ is only temperature-dependent and is not affected by the concentration of sulfuric acid (Equation 9). In addition, Figure 5b shows that


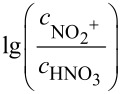


increases with the increase in sulfuric acid concentration when the temperature is fixed. In contrast, *nM*_c_ is a negative value that decreases with higher sulfuric acid concentration (Table S1 in Supporting Information File 1). As the concentration of sulfuric acid increases, the decrease in *nM*_c_ gradually surpassed the increase in


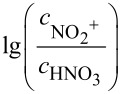


when the sulfuric acid concentration exceeded 94%, resulting in an overall decrease of *k* (Figure 7a). Similar trends were reported in the nitration of nitrobenzene [40] and *o*-nitrotoluene [18], suggesting that the phenomenon observed in our study is not isolated.

### Validation, extrapolation, and optimization

To validate the kinetic model and assess its ability to extrapolate, we conducted 18 experiments varying three residence times, three reaction temperatures, and two sulfuric acid concentrations. We then compared the theoretical and experimental values of conversion rates under these conditions (Figure 7b and Table S2 in Supporting Information File 1). Notably, 16 of these experiments were performed with a residence time exceeding the upper limit of the model construction, 4.7 min. The results revealed a strong alignment between the predicted and experimental conversion rates, with an average discrepancy of less than 2%. The smallest error was observed with a 98% sulfuric acid concentration at 35 °C and a residence time of 8.0 min, where the theoretical and experimental values nearly matched. Conversely, the largest error was at 94% sulfuric acid concentration, 40 °C, and a residence time of 9.3 min, with theoretical and experimental values of 90% and 86%, respectively. Increasing the residence time to 12.36 min amplified the error to approximately 8% (Figure 7c). A similar increase in error with prolonged residence time was noted in Kappe et al.’s kinetic modeling of the Buchwald–Hartwig amination reaction [41], where the theoretical and experimental values diverged by 4.1% when the residence time increased from 0.5 min to 4.2 min.

Building on the model’s demonstrated ability to extrapolate at prolonged residence times, we performed additional experiments with the reaction temperature increased to 45 °C (Figure 7c and Table S3 in Supporting Information File 1). This temperature exceeds the highest temperature used during the initial development of the kinetic model, which was 40 °C. This further extrapolation led to a 10% error at a residence time of 13.7 min, inferring that it would be prudent to avoid increasing the temperature to 45 °C if the aim is to maintain the discrepancy between the model predicted and experimental conversion rates below 10%.

Based on the observations above, the optimized reaction conditions were obtained: the sulfuric acid mass fraction was 94%, the initial concentration of IO was 0.5 mol/L, the reaction temperature was 40 °C, the molar ratio was 4.4:1, and the reaction time was 12.36 min. Under these conditions, the experimentally measured conversion was 87.4%.

This study marks the first time that the intrinsic kinetics of this reaction have been reported and utilized to optimize the process of nitration of *O*-methylisouronium sulfate within a continuous flow device. The highly exothermic nature of nitration makes the conversion from batch to continuous flow significantly safer. Additionally, the optimization model demonstrates excellent scalability and can accurately predict reaction conversions, with errors not exceeding 4%, for residence times beyond the modeling range (extending from the initial 1–5 minutes to 5–12 minutes in validation experiments). Compared to the original patent [2], the reaction time has been significantly reduced from tens of minutes to hours to less than 20 minutes while maintaining a lower sulfuric acid mass fraction and achieving higher conversion rates. Furthermore, the process does not require low temperatures, thereby reducing energy consumption and simplifying operation.

## Conclusion

In this work, a homogeneous nitration system for the synthesis of *O*-methyl-*N*-nitroisourea was constructed. To eliminate the mass transfer resistance between the two liquid phases during the reaction, a homemade simple and effective static mixer was used which rapidly achieved thorough mixing of the two phases with little temperature fluctuation. The effects of temperature, residence time, and sulfuric acid mass fraction on the reaction were investigated as well as the apparent and intrinsic rate constants based on nitric acid and NO_2_^+^ observations were obtained, respectively. The apparent rate constants observed based on nitric acid are highly correlated with the mass fraction of sulfuric acid, increasing and then decreasing as the mass fraction of sulfuric acid increases, with 94% sulfuric acid being the turning point. This is the result of a combination of the intrinsic rate constant, the sulfuric acid activity coefficient function, and the NO_2_^+^ concentration. Thus, the effect of different sulfuric acid mass fractions and temperatures on the apparent rate constants can be understood. In addition, a complete kinetic model of IO nitration based on NO_2_^+^ was developed to describe the reaction process, the activation energy of the IO nitration was calculated to be 192.57 kJ/mol. Furthermore, the accuracy of the kinetic model was verified by comparing the predicted data with the experimental data. Finally, the reaction was optimized by kinetic modeling and 87.4% conversion of IO was achieved under optimum conditions. This kinetic model can be used to understand the nitration process of IO and optimize the reactor design, which can serve as guidance for industrial production.

## Experimental

### Materials and methods

#### Chemicals

*O*-Methylisouronium sulfate (IO, 95%) was purchased from Shanghai Yien Chemical Technology Co., Ltd; fuming nitric acid (HNO_3_, 98.0%) was purchased from Sinopharm Chemical Reagent Co., Ltd.; sulfuric acid (H_2_SO_4_, 98.0%) was purchased from Sinopharm Chemical Reagent Co., Ltd.; pure water from AR, Hangzhou Wahaha Group Co., Ltd.; all reagents were used without further purification. Sulfuric acid solutions of different mass fractions were prepared with pure water and 98% concentrated sulfuric acid in an ice bath with stirring.

Solution A (IO): IO (0.1 mol, 24.64 g) was dissolved in H_2_SO_4_ (100 mL) under stirring conditions in an ice bath, solution volume *V*_A_ = 118 mL.

Solution B (H_2_SO_4_ + HNO_3_): HNO_3_ (0.44 mol, 18.49 mL) was dissolved in H_2_SO_4_ (100 mL) under stirring conditions in an ice bath, solution volume *V*_B_ = 112 mL.

#### Continuous flow microreactor system

The continuous flow microreactor system is shown in Figure 1. Solutions A and B were stored in two glass vials (500 mL) with lids and were preheated by two high-pressure PTFE pumps (pump A, pump B, JJRZ-10004F, Hangzhou JingJin Technology Co., Ltd.) and pumped into coiled stainless steel capillary tubes (SS316L, 1/16-inch diameter) that were sufficiently long (1 m). After being preheated to reaction temperature, the material was first initially mixed in a T-mixer (SS316L, 1/16-inch diameter), followed by a homemade static mixer at the outlet of the T-mixer to fully mix the material. The reaction coil (SS316L, 1/8-inch diameter) was connected directly to the outlet of the homemade static mixer, nitration took place in the reaction coil. The residence time was precisely controlled by changing the flow rate of the reaction mixture or the length of the reaction coil. All preheat tubes, mixers, and reaction coils were immersed in the same water bath to maintain a constant temperature. Finally, after controlling the residence time, the reaction was terminated by pumping excess pure ice water through a high-pressure PTFE pump (Pump C, JJRZ-10004F, Hangzhou Jingjin Technology Co., Ltd.) into the second T-mixer.

The homemade static mixer consisted of two different mixing units as shown in Figure 2b (total internal volume: 1.3154 mL). The first mixing unit consists of a section of stainless steel coil (SS316L, 1/16-inch diameter, Beijing Xiongchuan Technology Co. Ltd.) and an electronic thermometer (Beijing Xiongchuan Technology Co. Ltd.). The second mixing unit consisted of a section of PTFE piping filled with SiO_2_ beads (SiO_2_ beads, 3 mm diameter; piping, 1/4-inch diameter,10 cm length, Wuxi Hongxin Special Material Technology Co.) and an electronic thermometer connected to the outlet.

#### Sample analysis

When the continuous flow system was operated at steady state (after 2–3 times the residence time), the reaction solution was quenched and diluted by a large amount of ice water at the outlet of the reaction system. The quenched and diluted reaction solution was collected and analyzed by high-performance liquid chromatography (HPLC, ThermoFisher Ulcel3000), and the conversion of the samples was derived from the external standard method based on the regression equation of the HPLC standard curve. HPLC detection conditions: C18 column (10 μm, 4.6 × 250 mm, Welch Materials Shanghai, China), the mobile phase was 80% MeOH and 20% ultrapure water at a flow rate of 1 mL/min, and the detection wavelength was 195 nm. The conversion of IO was calculated by the following equation:


[14] $$x_{\text{IO}}=\left(1-\frac{c_{\text{IO}}}{c_{\text{IO}}+c_{\text{NIO}}}\right)$$

The residence time was calculated as follows:


[15] $$t=\frac{V}{Q_{\text{IO}}+Q_{{\text{HNO}}_{3}}}$$

where *t* is the reaction residence time and *V* is the volume of the microchannel. *Q*_IO_ and *Q*_HNO3_ are the volume flow rates of the raw material aqueous solution, respectively. Samples were tested three times under the same conditions and averaged to minimize errors.

#### Kinetic modeling optimization process

The classical integral method was employed to determine the reaction order [42]. Various integral forms of kinetic equations corresponding to different reaction orders were fitted against the experimental data. The reaction orders yielding the highest R^2^ were selected as the best fit. Subsequently, the least squares method was used to fit the kinetic data obtained under different reaction conditions, allowing for the determination of the pre-exponential factors and activation energies. Finally, the accuracy of the resulting kinetic model was validated through experimental testing.

## Supporting Information

File 1 Additional information.

## Data Availability

All data that supports the findings of this study is available in the published article and/or the supporting information to this article.

## References

[1] Elbert A, Haas M, Springer B, Thielert W, Nauen R (2008). Pest Manage Sci.

[2] Uneme H, Konobe M, Ishizuka H, Kamiya Y (1997). Preparation of heteroarylmethylisoureas and related compounds. WO Patent.

[3] Uneme H, Kamiya Y, Konobe M, Yamada J (1999). Manufacture of N-(heterocyclylmethyl)-N'-nitroisoureas. WO Patent.

[4] Brady J F, Simmons D P, Wilson T E (2001). Immunoassay for neonicotinoid insecticides. WO Patent.

[5] Köckinger M, Wyler B, Aellig C, Roberge D M, Hone C A, Kappe C O (2020). Org Process Res Dev.

[6] Magosso M, van den Berg M, van der Schaaf J (2021). React Chem Eng.

[7] Sheng L, Chen Y, Wang K, Deng J, Luo G (2021). Chem Eng Sci.

[8] Sheng L, Ma L, Chen Y, Deng J, Luo G (2022). Chem Eng J.

[9] Guo S, Zhan L-w, Li B-d (2023). Chem Eng J.

[10] Jin N, Song Y, Yue J, Wang Q, Lu P, Li Y, Zhao Y (2023). Chem Eng Sci.

[11] Rahaman M, Mandal B P, Ghosh P (2007). AIChE J.

[12] Taylor C J, Pomberger A, Felton K C, Grainger R, Barecka M, Chamberlain T W, Bourne R A, Johnson C N, Lapkin A A (2023). Chem Rev.

[13] Taylor C J, Booth M, Manson J A, Willis M J, Clemens G, Taylor B A, Chamberlain T W, Bourne R A (2021). Chem Eng J.

[14] Burés J, Larrosa I (2023). Nature.

[15] Yao Z, Xu X, Dong Y, Liu X, Yuan B, Wang K, Cao K, Luo G (2020). Chem Eng Sci.

[16] Hughes E D, Ingold C K, Reed R I (1946). Nature.

[17] Cui Y, Song J, Du C, Deng J, Luo G (2022). AIChE J.

[18] Song J, Cui Y, Luo G, Deng J, Wang Y (2022). React Chem Eng.

[19] Song J, Cui Y, Sheng L, Wang Y, Du C, Deng J, Luo G (2022). Chem Eng Sci.

[20] Zhang S, Zhu C, Feng H, Fu T, Ma Y (2021). Chem Eng Sci.

[21] Holvey C P, Roberge D M, Gottsponer M, Kockmann N, Macchi A (2011). Chem Eng Process.

[22] Tajik Ghanbari T, Rahimi M, Ranjbar A A, Pahamli Y, Torbatinejad A (2023). Phys Fluids.

[23] Al-Azzawi M, Mjalli F S, Husain A, Al-Dahhan M (2021). Ind Eng Chem Res.

[24] Bhagat A A S, Peterson E T K, Papautsky I (2007). J Micromech Microeng.

[25] Su Y, Chen G, Yuan Q (2011). Chem Eng Sci.

[26] Dunstan A E (1914). Proc Chem Soc, London.

[27] Dean W R, Hurst J M (1959). Mathematika.

[28] Jonas Bolinder C, Sundén B (1995). Exp Therm Fluid Sci.

[29] Lü Y, Zhu S, Wang K, Luo G (2016). Chin J Chem Eng.

[30] Tata Rao L, Goel S, Kumar Dubey S, Javed A (2019). J Phys: Conf Ser.

[31] Bazargan-Lari Y, Movahed S, Mashhoodi M (2017). J Mech.

[32] Li S, Zhang X, Ji D, Wang Q, Jin N, Zhao Y (2022). Chem Eng Sci.

[33] Yang M, Liao C, Tang C, Zhang P, Huang Z, Li J (2021). Phys Chem Chem Phys.

[34] Olah G A, Malhotra R, Narang S C (1989). NITRATION: Methods and Mechanisms. Across Conventional Lines.

[35] Marziano N C, Tomasin A, Traverso P G (1981). J Chem Soc, Perkin Trans 2.

[36] Deno N C, Peterson H J, Sacher E (1961). J Phys Chem.

[37] Marziano N C, Tomasin A, Tortato C, Zaldivar J M (1998). J Chem Soc, Perkin Trans 2.

[38] Ross D S, Kuhlmann K F, Malhotra R (1983). J Am Chem Soc.

[39] Wen Z, Yang M, Zhao S, Zhou F, Chen G (2018). React Chem Eng.

[40] Rahaman M, Mandal B, Ghosh P (2010). AIChE J.

[41] Wagner F, Sagmeister P, Jusner C E, Tampone T G, Manee V, Buono F G, Williams J D, Kappe C O (2024). Adv Sci.

[42] Xu Q, Fan H, Yao H, Wang D, Yu H, Chen B, Yu Z, Su W (2020). Chem Eng J.

