# Supplementary material for: Homogeneous continuous flow nitration of O-methylisouronium sulfate and its optimization by kinetic modeling
Source: Beilstein J Org Chem. 2024 Sep 24;20:2408–20. doi: 10.3762/bjoc.20.205 (PMC11445654; doi:10.3762/bjoc.20.205)
Supplement: File 1 — Additional information. [file Beilstein_J_Org_Chem-20-2408-s001.pdf]

BEILSTEIN JOURNAL OF ORGANIC CHEMISTRY

Supporting Information

for

Homogeneous continuous flow nitration of *O*-methylisouronium sulfate and its optimization by kinetic modeling

Jiapeng Guo, Weike Su and An Su

Beilstein J. Org. Chem. **2024,** *20,* 2408–2420. doi:10.3762/bjoc.20.205

Additional information

License and Terms: This is a supporting information file under the terms of the Creative Commons Attribution License (https://creativecommons.org/licenses/by/4.0). Please note that the reuse, redistribution and reproduction in particular requires that the author(s) and source are credited and that individual graphics may be subject to special legal provisions.
The license is subject to the *Beilstein Journal of Organic Chemistry* terms and conditions: (https://www.beilstein-journals.org/bjoc/terms)

Part 1. Effect of molar ratio

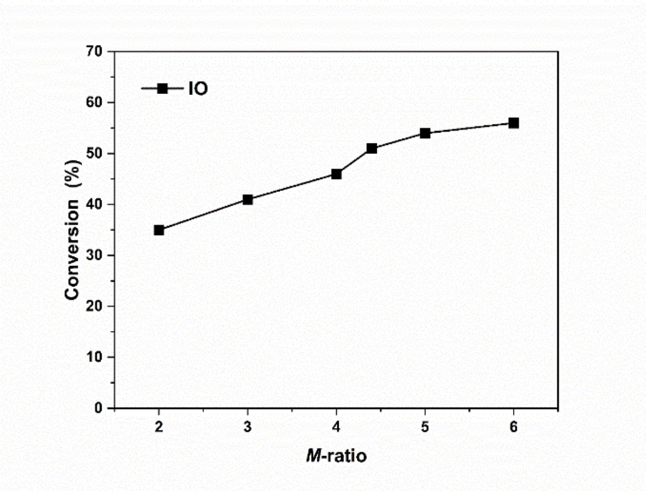

Figure S1: Variation of NIO conversion with HNO_3 molar ratio. The conversion was determined by high performance liquid chromatography (HPLC).

Part 2. Determination of the reaction orders

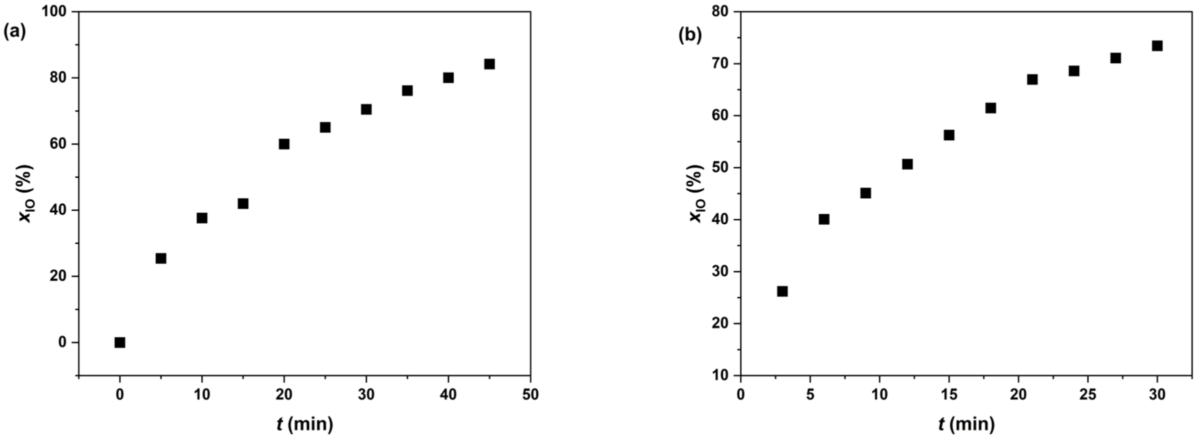

Figure S2: (a) x_{IO} versus reaction time (t). (b) x_{IO} versus reaction time t. Determination of IO reaction order conditions: reaction temperature (T) = 0 °C; initial concentration of reactants in reaction mixture: $c_{\mathrm{IO}0}$ = 1 mol/L, $c_{\mathrm{HNO_3}0}$ = 15 mol/L. Determination of HNO_3 reaction order conditions: reaction temperature (T) = 10 °C; initial concentration of reactants in reaction mixture: $c_{\mathrm{IO}0}$ = 1 mol/L, $c_{\mathrm{HNO_3}0}$ = 4.4 mol/L. The conversion was determined by high performance liquid chromatography (HPLC).

Part 3. Apparent reaction rate

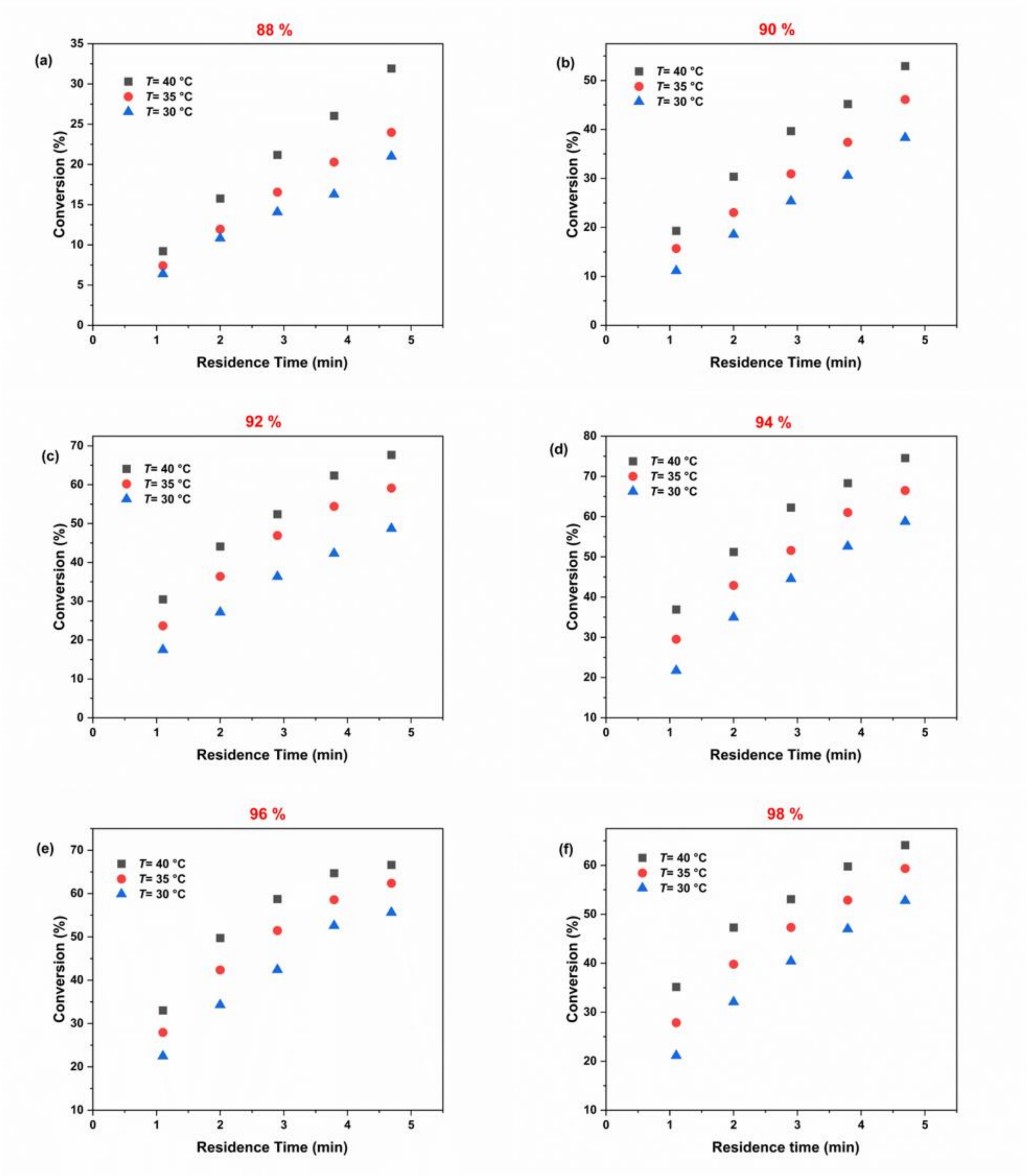

Figure S3: Variation of x_{IO} with t at different temperatures (30 °C, 35 °C, 40 °C) and different H_2SO_4 mass fractions (88%, 90%, 92%, 94%, 96%, 98%).

Part 4. Determination of M_C function

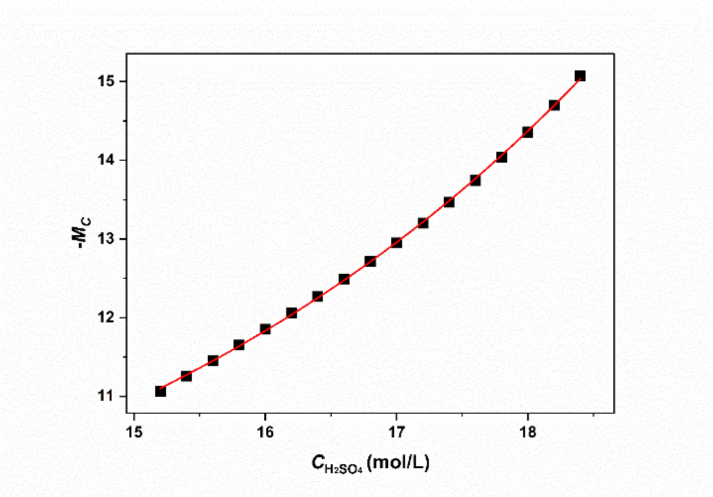

Figure S4: The results of polynomial fitting the data of M_c function at 298K.[1]

Part 5. Values of nM_c and $\lg\left(\frac{c_{NO_2^+}}{c_{HNO_3}}\right) + \lg k_0$ under different temperatures and H_2SO_4 mass fraction

Table S1: Values of nM_c and $\lg\left(\frac{c_{NO_2^+}}{c_{HNO_3}}\right) + \lg k_0$ at different temperatures and H_2SO_4 mass fraction.

Mass fraction of H_2SO_4 (wt %)	Temperature (°C)	$\lg\left(\frac{c_{NO_2^+}}{c_{HNO_3}}\right) + \lg k_0$	nM_c
88	30	11.285	-12.931
	35	11.705	-13.230
	40	12.226	-13.592
90	30	12.015	-13.275
	35	12.431	-13.591
	40	12.947	-14.023
92	30	12.745	-13.871
	35	13.156	-14.146
	40	13.668	-14.567
94	30	13.476	-14.462
	35	13.882	-14.791
	40	14.389	-15.209
96	30	14.206	-15.225
	35	14.607	-15.572
	40	15.110	-16.053
98	30	14.936	-16.026
	35	15.333	-16.393
	40	15.831	-16.859

Part 6. Kinetic model validation experiments under different conditions

Table S2: Kinetic model validation experiments under different conditions

Mass fraction of H_2SO_4 (wt %)	Temperature (°C)	Time (min)	Theoretical conversion (%)	Actual conversion (%)	Errors (%)
94	40	3.5	60	62	2
		6.3	80	81	1
		9.3	90	86	4
	35	4.2	60	62	2
		5.6	70	71	1
		7.6	80	79	1
	30	5.1	60	61	1
		6.8	70	69	1
		9.3	80	78	2
98	40	5.6	60	62	2
		7.4	70	72	2
		10.2	80	78	2
	35	6.0	60	59	1
		8.0	70	70	0
		11.0	80	77	3
	30	6.4	60	61	1
		8.6	70	69	1
		11.7	80	77	3

Part 7. Reaction optimization using kinetic models

Table S3: Reaction optimization using kinetic models.

**Mass fraction of H_2SO_4 (wt %)**	**Temperature (°C)**	**Time (min)**	**Theoretical conversion (%)**	**Actual conversion (%)**	**Errors (%)**
94	45	7.8	90	83.9	6
		10.3	95	85.8	9
		13.7	98	87.6	10
	40	7.6	85	81	4
		12.4	95	87.4	8

References

1. Marziano, N. C.; Tomasin, A.; Traverso, P. G. *Journal of the Chemical Society, Perkin Transactions 2* **1981**, (7), 1070-1075, 10.1039/P29810001070.
